# Supplementary material for: Biologics in Severe Eosinophilic Asthma: Three-Year Follow-Up in a SANI Single Center
Source: Biomedicines. 2022 Jan 18;10(2):200. doi: 10.3390/biomedicines10020200 (PMC8869384; doi:10.3390/biomedicines10020200)
Supplement: Supplementary file 1 [file biomedicines-10-00200-s001.zip › biomedicines-1555042-supplementary.pdf]

a. FEV1 (% of predicted value)

Responders		Non-responders	
T0 (68 patients)	78.8 ± 19.7	T0 (22 patients)	70.9 ± 20.4
T1 (68 patients)	86.8 ± 19.7	T1 (22 patients)	77.8 ± 17.4
T2 (55 patients)	87.7 ± 19.1	T2 (18 patients)	73.6 ± 16.5
T3 (36 patients)	90.8 ± 19.5	T3 (17 patients)	83.6 ± 17.7
T4 (25 patients)	85.9 ± 18.4	T4 (14 patients)	76.4 ± 19.7

		p			p
T0	T1	n.s.	T0	T1	n.s.
	T2	n.s.		T2	n.s.
	T3	0.002		T3	0.001
	T4	n.s.		T4	n.s.

b. F_ENO (ppb)

Responders		Non-responders	
T0 (68 patients)	46.4 ± 30.9	T0 (22 patients)	42.9 ± 25.9
T1 (68 patients)	33.9 ± 23.3	T1 (22 patients)	39.9 ± 27.2
T2 (55 patients)	29.6 ± 23.3	T2 (18 patients)	37.3 ± 28.2
T3 (36 patients)	23.2 ± 18.1	T3 (17 patients)	34.1 ± 27.3
T4 (25 patients)	19.1 ± 10.5	T4 (14 patients)	21.3 ± 12.6

		p			p
T0	T1	0.043	T0	T1	n.s.
	T2	0.035		T2	n.s.
	T3	0.008		T3	n.s.
	T4	<0.001		T4	n.s.

Table S1 *– Trends of the analyzed variables over time in responders and non-responders, comparisons with baseline. Panel a: FEV1 (% of predicted value); Panel b:- F_ENO (ppb).*
